# Time to take HPV infection in colorectal cancer patients more seriously

**DOI:** 10.3389/fmed.2024.1418359

**Published:** 2024-07-10

**Authors:** Mahsa Javadi, Shahram Jalilian, Malek Kanani, Vahid Kia, Abdolhassan Talaiezadeh, Kambiz Ahmadi Angali, Mohammad Karimi Baba Ahmadi, Manoochehr Makvandi

**Affiliations:** ^1^Infectious and Tropical Diseases Research Center, Health Research Institute, Ahvaz Jundishapur University of Medical Sciences, Ahvaz, Iran; ^2^Virology Department, School of Medicine, Ahvaz Jundishapur University of Medical Sciences, Ahvaz, Iran; ^3^Department of Pathology, Imam Khomeini Hospital, Ahvaz Jundishapur University of Medical Sciences, Ahvaz, Iran; ^4^Department of Medical Biotechnology, School of Medicine, Shahroud University of Medical Sciences, Shahroud, Iran; ^5^Department of Biochemistry, Faculty of Medicine and Dentistry, University of Alberta, Edmonton, AB, Canada; ^6^Cancer, Research Center, Ahvaz Jundishapur University of Medical Sciences, Ahvaz, Iran; ^7^Department of Biostatistics and Epidemiology, School of Public Health, Ahvaz Jundishapur University of Medical Sciences, Ahvaz, Iran; ^8^Department of Medical Biotechnology, School of Medicine, Ahvaz Jundishapur University of Medical Sciences, Ahvaz, Iran

**Keywords:** Human Papillomavirus, colorectal cancer, E6 protein, E7 protein, urine, exosome

## Abstract

**Background:**

The association between viral infections and colorectal cancer (CRC) remains an enigma in cancer research. Certain types of Human Papillomaviruses (hr-HPVs), known for their oncogenic properties, have been observed in particular CRC biopsies, further adding to the enigma surrounding this association.

**Materials and methods:**

This cross-sectional study was conducted on 40 confirmed cases of CRC adenocarcinoma. The presence and genotyping of HPV DNA in colorectal fresh tissue and urine samples was assessed using an HPV DNA hybridization kit. A subset of serum samples from both CRC cases and healthy volunteers was randomly chosen and subjected to western blot to investigate the presence of HPV16 E6/E7 oncoproteins carried by exosomes.

**Results:**

It was observed that 26/40 HPV-positive CRC patients demonstrated 7 times more chance to develop colorectal cancer when compared to those 8/40 normal tissue (odds ratio [OR] = 7.4; confidence interval [CI] 95% = 0.483156–0.793718; *p* < 0.001). Of 26 HPV-positive CRC patients, 14 urine samples were also showed HPV DNA positivity (*p* = 0.013). High-risk HPV16 was the most prevalent genotype detected in both 24/40 tumor and 12/40 urine samples (*p* < 0.001). The tumor sample of a male was HPV45, while another male’s urine sample was HPV31. A female CRC patient had HPV83 in tumor and HPV56 in urine. Here, was the first detection of HPV83 in a CRC patient. Notably among 20 randomly selected serum exosome samples, one serum sample concurrently tested positive for both HPV16 E6 and E7 oncoproteins, and one sample tested positive for HPV16 E7 oncoprotein.

**Conclusion:**

High risk HPV DNA detection in CRC urine samples supports non-invasive screening tools. Detection of HPV16 E6 and E7 oncoproteins in exosomes from serum samples shows potential for non-invasive diagnostics. HPV’s potential role in CRC development is also underscored. HPV vaccination should be implemented in low- and middle-income countries to prevent cancer.

## Introduction

Colorectal cancer (CRC) is recognized as one of the most prevalent malignancies worldwide. As documented by the GLOBOCAN database, the year 2020 witnessed an estimated 1.9 million new cases of colorectal cancer, including the anal cancer, with approximately 935,000 fatalities (1). In Iran, the estimated incidence of colorectal cancer in 2020 was 9.1%, positioning it as the third most prevalent cancer after breast and stomach cancer (2).

Colorectal cancer is characterized as a multifactorial disease. Key contributing factors encompass familial adenomatous polyposis (FAP), inflammatory bowel disease (IBD), elevated body mass index (BMI ≥ 25), human development index (HDI) and lifestyle (1, 3, 4). Age, smoking, alcohol consumption, and certain comorbidities like diabetes further contribute to the intricate spectrum of potential risk factors associated with colorectal cancer (1, 5).

Currently, alongside genetic and environmental factors, there is a growing consesus among scientists on the plausible direct and indirect role of microbial infections in the pathogenesis of colorectal cancer. Among these microbial agents, the high-risk Human Papillomaviruses (hr-HPVs) are particularly noteworthy as they represent one of the most prevalent sexually transmitted infections involving anogenital region (6). HPVs are primarily transmitted through skin-to-skin or skin-to-mucosa contact, however HPV genomes have been identified in various bodily and environmental samples including, blood, urine, feces, amniotic fluid, sewage water, gynecological equipment and pool water (7). These agents possess oncoproteins E6 and E7 with definitive impact on the activation of cellular proto-oncogenes (P53 and pRb) (6).

HPVs show high resistance to heat, drying, and certain disinfectants like glutaraldehyde (GTA) and ortho-phthaladehyde (OPA), while hydrogen peroxide (Trophon^®^ EPR) EPR and hypochlorite effectively disinfect against HPV 16 and HPV 18 (8).

The direct role of high-risk HPVs in cervical cancer (CC) or other anogenital cancers and Head and Neck Squamous Cell Carcinoma (HNSCC) is established and well-documented (6). Most genital area cancers are caused by HPVs including cervical, penile and even anal cancers. HPVs are considered as significant risk factor for esophageal, gastric, liver, colorectal and urinary bladder cancers as well (6). The clinical manifestation of HPV infection differs in sex organs between men and women, with men often not showing symptoms but acting as carriers to their sexual partners (9).

The initial report suggesting a potential correlation between HPV infection and CRC dates back to 1990, by of Kirgan et al. (9). Subsequent investigations have successfully detected HPV in colon and rectum tissue using molecular or histopathological techniques (10–13). Some studies postulate that HPVs may also disseminate to distant organs via lymphatic ducts, potentially inducing to critical states of metastasis and unfavorable prognosis (14).

Extracellular vesicles, including exosomes, ranging from 30 to 150 nm have emerged as objects of investigation in the field of cancer, along with infectious agents. Lipid bilayer exosomes release by nearly every tissue and cell type. These low immunogenic cell-derived compartments encapsulate and transport a diverse range of molecules, including proteins and microRNAs, providing insights into the host cell’s status (15). The dysregulated secretion of exosomes in cancer cells leads to an elevated production that may contain cancer-related factors and contribute to tumor-cell invasion (16, 17). Beyond cellular factors, exosomes, functioning as vehicles have the capacity to accommodate viral components, a complete genome, or even an intact virion itself as a cargo. This unique characteristic has prompted researchers to designate exosomes as potential candidates for liquid biopsy candidates (18, 19).

The present study aims to investigate the potential link between the Human Papillomavirus and colorectal cancer development, while exploring the possible presence of HPV DNA in urine and HPV oncoproteins E6 and E7 transmission by exosomes through bloodstream.

## Materials and methods

### Study population

This cross-sectional study included a total of 40 individuals, 21 female and 19 male patients with ages ranging 37 to 86 years old, referred to Apadana Hospital of Ahvaz for surgical treatment. The enrollment criteria for this study required the presence of “confirmed adenocarcinoma” at any histopathological stage. The adenocarcinoma had been confirmed by colonoscopy, sonography and histopathological confirmation. No ethnicity was evaluated. From February 2021 to February 2022, fresh colorectal cancer biopsies, including both the tumor and paired adjacent non-cancer tissue (located 15–20 cm apart from the tumor), were obtained by a surgeon after the removal of cancer organ. The aseptic conical microtube 1.5 was employed for the collection of tumor and normal tissue specimens. Each microtube, containing 400 μL of RNA stabilization buffer (DNA biotech, Iran), was utilized for soaking approximately 30 to 40 mg tissue of the colorectal cancer (CRC) patients. Subsequently, all specimens were promptly transferred to the virology laboratory under cold chain conditions within a period of 30 min and stored at −20°C for subsequent analysis. In addition to tissue biopsy, a serum sample was also obtained from each participant along with 10 serum samples from healthy volunteers as controls for exosome investigation. These samples underwent centrifugation and the resulting supernatants immediately stored at -80°C to preserve their molecular integrity. Urine samples –to avoid genital contamination- were collected from newly installed urine bags and immediately processed. These samples underwent centrifugation, and the resulting precipitates were promptly stored at −20°C.

### DNA extraction from biopsies

The DNA extraction process employed an in-house phenol/chloroform method. To ensure safety and prevent contamination all the procedures for DNA extraction were performed in a biological class II safety cabinet. Initially, samples were subjected to digestion at 55°C overnight with the addition of 400 μL lysis buffer, consisting of Tris–HCL 10 mM, EDTA 100 mM, NaCl 50 mM, and SDS 0.5%. To enhance digestion, a thermal shock was administered. During this step, samples were placed at -80°C for 1 hour followed by immediate transfer to a 94°C heater and vortexing. Subsequently, supernatants were transferred to a new microtube with an equal volume of phenol (pH = 8), gently mixed and centrifuged for 6 min at 3500 g. Supernatants were then transferred to another microtube with an equal volume of phenol/chloroform/isoamyl alcohol (25:24:1) (Sigma-Aldrich, Co). After 6 minutes of gentle mixing and centrifugation at 3500 g, supernatants were transferred to the final microtube, and ice-cold 96% ethanol was added twice the volume. Microtubes were centrifuged for 30 min at 17000 g. Following the addition of 100 μL ice-cold 70% ethanol, the resulting pellet was centrifuged for 30 min at 17000 g, dried, and resuspended in 30 μL of distilled water. The concentration and purity of diluted DNA precipitates were evaluated using a Nanodrop spectrophotometer. The extracted DNAs were stored at -20°C.

### Urine DNA extraction

Urine samples (15–20 milliliters each) underwent centrifugation at 1400 g for 25 min. Subsequently, a salting-out procedure was employed as an in-house DNA extraction method. The cell pellet was treated with a lysis buffer consisting of TES (500 μL), SDS (20 μL), and proteinase K (25 μL), with a 2 h of incubation/vortexing at 65°C. The addition of 220 μL ice-cold 5 M NaCl, was followed by a 5 min shaking step, and a 20 min centrifugation at 3500 g. After transferring the supernatant to a new microtube, 550 μL of 100% ice-cold isopropanol was added. Microtubes were centrifuged at 17000 g for 5 min. Subsequently, 100 μL of ice-cold 70% ethanol was added, and the resulting pellet was centrifuged at 17000 g for 5 min. The dried DNA samples were resuspended in 30 μL of TE, and the DNA samples were stored at −20°C.

### Genotyping

HPV genotyping was performed using the Opegen^®^ hybridization kit (Operon/Spain), enabling the detection of 37 HPV genotypes (18 low-risk and 19 high to medium risk). The PCR products that were not detectable by hybridization technique were sequenced using Sanger sequencing.

### Exosome extraction

Venous blood samples were obtained in 10 mL venoject tubes from both CRC patients and non-CRC volunteers. Immediately upon collection, all blood samples underwent centrifugation at 500 g for 10 min, and the sera were collected and centrifuged at 2,000 g at 4°C for 30 min. The supernatants were carefully transferred to a new tube without pellet contamination and underwent an additional centrifugation step at 12,000 g at 4°C for 45 min. Following this, the supernatants were meticulously transferred to ultracentrifuge tubes without pellet contamination and centrifuged with Beckman ultracentrifuge rotor type 1,202 (k-factor: 204) at 64,000 g at 4°C for 3 h to pellet exosomes. Subsequently, supernatants were gently discarded and the pelleted exosomes were resuspended in 1.5 mL ice-cold PBS, filtered through 0.22 μm filters and subjected to a second round of ultracentrifugation at 64,000 g at 4°C for 3 h. After discarding the supernatant, the exosome pellets were resuspended in 100 μL PBS and stored at -80°C. The purified exosomes were sent to Razi Metallurgical Research Center, Karaj city, Iran to check the verification of purity of exosome particles by scanning electron microscopy (SEM) and transmission electron microscopy (TEM). Western blot analysis using CD63 monoclonal antibody (GTX41877, GeneTex, United States at 1:500. dilution) was performed for exosomes extraction verification.

### Western blot

The protein concentration of the isolated exosome pellets was determined using an in-house Bradford assay. In Brief, bovine serum albumin (BSA) standards and samples were mixed with 1 mL of bradford working reagent and the absorbance was measured at 595 nm. The obtained exosome pellets were mixed with an equal volume of 2X Laemmli sample buffer and heated at 94°C for 5 min. Samples were then loaded onto a polyacrylamide gel and total protein extracts were separated using 12% sodium dodecyl sulfate–polyacrylamide gel electrophoresis (PAGE) (Bio-Rad, CA, United States). Then, the gel was transferred to PVDF membranes (Thermo Fisher Scientific) and blocked with 5% skimmed milk powder in TBST (20 mM TBS; pH 8.6, 150 mM NaCl and 0.1% Tween 20) for 1 h at room temperature. Mouse primary monoclonal antibodies against E6 protein and E7 protein of papillomavirus 16 (1:1500 dilution, Santa Cruz Biotechnology, Santa Cruz, CA, United States) were incubated with the membranes overnight at 4°C. Subsequently, the membranes were washed with TBST three times and then incubated with Goat Anti-Mouse IgG1 (HRP) secondary antibody (Cat No: ab97240; Abcam). The protein bands were then identified using chemiluminescence method (ECL).

### Statistical analysis

Data analysis was performed using SPSS software version 24.0. Descriptive statistics and mean ± standard deviation were reported for categorical and numerical data. Chi-squared and/or Fisher’s exact test compared frequencies between tumor HPV positive group and other parameters. Significance level was set at ≤0.05.

### Ethical considerations

This study was conducted following approval ethical code IR.AJUMS.REC.1399.817 by the ethics committee of Ahvaz Jundishapur University of Medical Sciences, Ahvaz, Iran. Prior to sample collection, informed consent was obtained from patients or their corresponding relatives.

## Results

Forty freshly obtained colorectal tumor specimens and forty corresponding adjacent tissues were subjected to investigation for viral DNA. The mean age of the patients was 62.4 ± 12.7 years ranging from 37 to 86 years. In the current study, an earlier onset of CRC was observed, with the youngest patient being 37 years old who his tumor and urine were both tested positive for HPV16. HPV DNA was identified in 26/40 (65%) colorectal tumor specimens and 8/40 (20%) adjacent tissues. It was observed that the HPV-positive CRC patients demonstrated 7 times more chance to develop colorectal cancer comparing to the adjacent normal tissue (odds ratio [OR] = 7.4; confidence interval [CI] 95% = 0.483156–0.793718; *p* < 0.001). Out of 26 HPV positive samples, 21 were female and 19 were male participants (*p* = 0.54). Of 26/40 tumor HPV-positive patients, 14 urine samples were also showed HPV DNA positivity (*p* = 0.013). The corresponding tumor sample of just two HPV-positive urines were HPV-negative. A high frequency of high-risk HPV type 16 among tumor samples 24/26 (92%) and urine samples 12/14 (86%) was observed (*p* < 0.001). Among 24 CRC patients positive for HPV16, 13/21 were females and 11/19 were males (*p* = 0.79). A few number of samples contained HPV types other than type 16. A tumor sample from a male patient tested HPV45, while another male’s urine sample was HPV31. A female CRC patient had HPV83 in tumor and HPV56 in urine. No instances of HPV genotypes co-infection were detected in the colorectal tissue and urine samples. The quantity of HPV16 positive tumor samples showed no significant differences between genders. Positive HPV frequency in urine samples was 10/21 in women and 4/19 in men (*p* = 0.08). For urine samples, the HPV16 positivity rate in women (9/21 samples) compare with in men (3/19 samples) was not significant (*p* = 0.062). Demographic and clinical factors, stratified by the presence or absence of HPV DNA, are detailed in Table 1.

**Table 1:** Demographic information of samples regarding the tumor HPV status.

Clinical or pathologic feature	Tumor HPV positive (%)	Tumor HPV negative (%)	*p*-value
Tumor			
Total = 40	26 (65%)	14 (35%)	<0.001
Age			0.41
≥60 = 28	19 (62%)	9 (38%)	
<60 = 12	7 (75%)	5 (25%)	
Gender			0.54
Female = 21	14 (67%)	7 (33%)	
Male = 19	12 (63%)	7 (37%)	
Health conditions			
DM = 11	8 (73%)	3 (27%)	0.40
HTN = 16	10 (63%)	6 (37%)	0.52
BMI ≥ 25 = 21	11 (52%)	10 (48%)	0.08
CEA			0.08
≥5 = 10	4 (40%)	6 (60%)	
Smoking/Drug = 7	6 (86%)	1 (14%)	0.21
Family History of any cancer = 12	8 (67%)	4 (33%)	0.60
Tumor location			0.54
Ascending colon = 7	5 (71%)	2 (29%)	
Descending colon = 4	3 (75%)	1 (25%)	
Sigmoid = 15	8 (53%)	7 (47%)	
Rectum = 10	8 (80%)	2 (20%)	
Cecum = 3	1 (33%)	2 (67%)	
Transverse colon = 1	1 (100%)	0 (0%)	
Pathology/Histology			
Adenocarcinoma = 40			0.42
Classic = 33	20 (61%)	13 (39%)	
Mucinous =6	5 (83%)	1 (17%)	
Signet ring cell =1	1 (100%)	0 (0%)	
Differentiation			0.78
Well differentiated =17	10 (59%)	7 (41%)	
Moderately differentiated =20	14 (70%)	6 (30%)	
Poorly differentiated =3	2 (67%)	1 (33%)	
Relapse/Metastasis =7	6 (86%)	1 (14%)	0.21
TNM classification			0.72
T1 = 5	3 (60%)	2 (40%)	
T2 = 9	5 (56%)	4 (44%)	
T3 = 18	13 (72%)	5 (28%)	
T4 = 6	3 (50%)	3 (50%)	
Cancer Stage			0.80
I = 11	7 (64%)	4 (36%)	
II = 13	9 (69%)	4 (31%)	
III = 12	7 (58%)	5 (42%)	
IV = 2	1 (50%)	1 (50%)	

DM, diabetes mellitus; HTN, hypertension; BMI, body mass index.

The prevalence of HPV DNA in tumor samples of patients with diabetes mellitus, hypertension (HTN) and smokers/drug users, was not found to be significant (Table 1). Among the 7 patients who experienced metastasis or relapse, 6 patients tested positive for HPV16 (*p* = 0.30). Other clinical findings, including tumor location, cancer staging, TNM classification, and histopathology also did not yield statistically significant results with HPV status.

**Figure 1:**
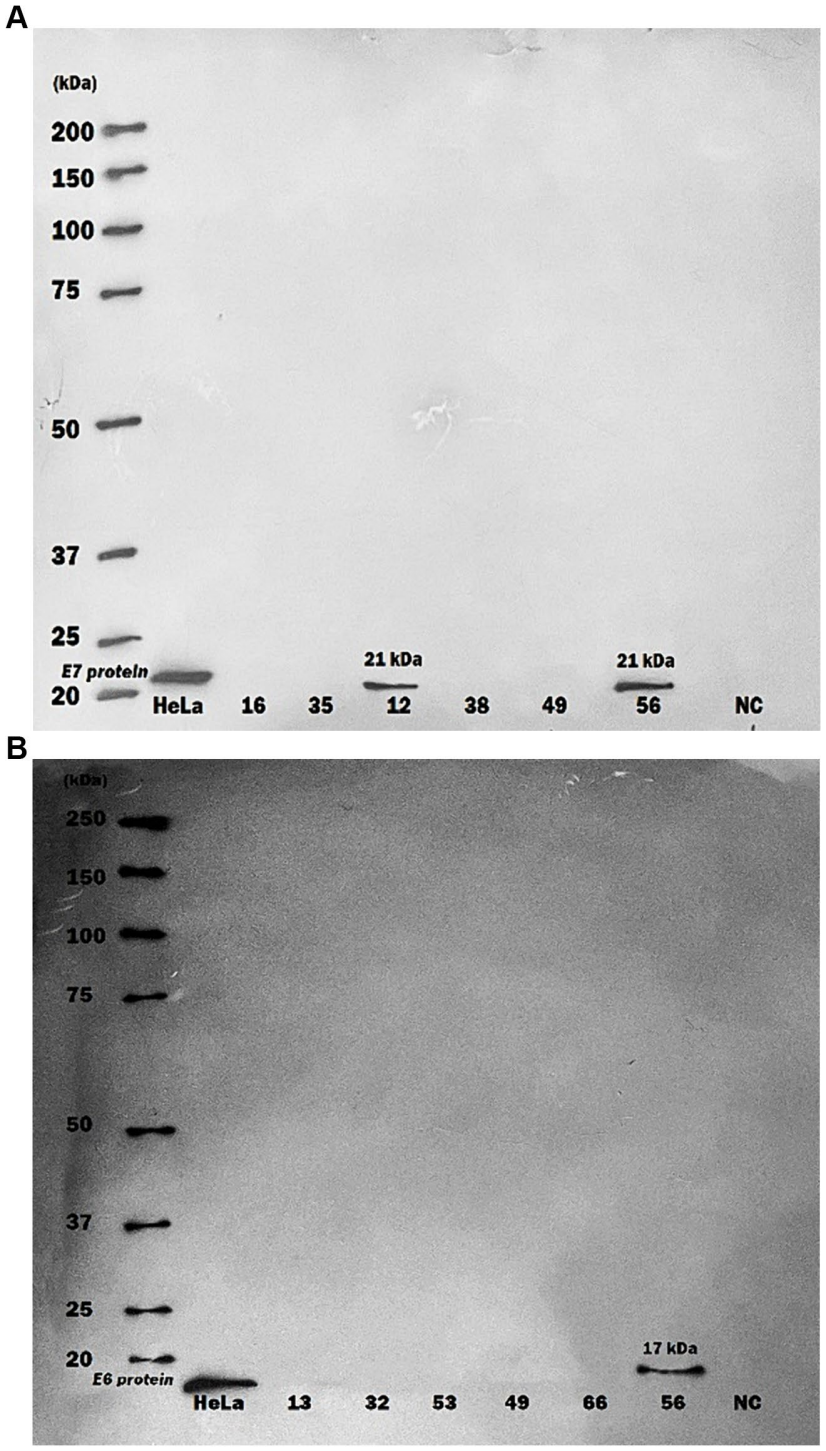
Western blot analysis of human papillomavirus 16 oncoproteins in purified serum exosome samples. Serum samples underwent multiple ultra-centrifugation steps to isolate purified exosomes. Exosome samples were then blotted on PVDF paper and protein detection was performed by using specific monoclonal antibodies against HPV16 E6 and E7 proteins. **(A)** A serum exosomes sample of one CRC patient showed only positive for HPV16 E7 protein (21 kDa) by Western blot, **(B)** A serum exosomes sample of one more CRC patient displayed positive for both HPV16 E6 (17 kDa) and E7 proteins. HeLa cells were used as Positive Control; NC, negative control.

In a different aspect of the research, HPV16 E6 and E7 oncoproteins were randomly examined within purified serum-exosome specimen using western blot analysis. Out of 20 randomly selected samples from CRC and non-CRC patients, two tested positive for HPV16. One showing both E6/E7 oncoproteins positivity while the other was positive for E7 protein only (Figure 1).

## Discussion

Colorectal cancer is the third diagnosed cancer in both sexes globally and the second leading cause of cancer-related deaths after lung cancer. Recent reports highlight an escalating incidence of CRC diagnoses among younger individuals. Considering this fact, the American Cancer Society revised its guidelines in 2018, recommending the initiation of screening at the age of 45 years (1). In the current study, an earlier onset of CRC was observed, with the youngest patient being 37 years old having HPV16-positive tumor and urine. Health policymakers should be promptly informed about this discovery. In recent years, several factors including alterations in diet, lifestyles, sedentary and obesity have been speculated to influence molecular and physiological characteristics resulting in the risk of CRCs and other cancer types in younger populations (20, 21).

Over 90 percent of CRC cases are diagnosed as adenocarcinomas and the major variants of adenocarcinoma are classified as classic adenocarcinoma. Considering previous data, classic adenocarcinoma with well to moderately differentiated cells are considered as the most prevalent colorectal cancers (22). In present study, classic adenocarcinoma and well to moderately differentiated adenocarcinomas were the most identified cases as well, accounting for 82 and 92%, respectively (*p* = 0.004). However, the frequency of HPV among these groups was not found to be statistically significant.

Several oncogenic infectious agents have been considered as potential contributors to CRC formation including hr-HPVs, EBV, JC virus, Fusobacterium spp. and CMV (21, 23, 24). However, given a degree of controversy surrounding their isolation, this relationship has not been yet proved for any of these microbial agents. This discrepancy for hr-HPVs in particular, may be attributed to factors such as small sample size, the geographical region of sampling, sample storage, individual or environmental hygienic conditions, sexual activity, and other related variables to influence HPV transmisission (25). In this study, the HPV genome with high frequency was observed in 65% of tumor tissues and 35% of urine samples. Among these, all but two samples of each sample series exhibited the presence of high-risk HPV type 16 (*p* < 0.001), which notably has the highest prevalence in most geographical regions worldwide as well (6). The other identified high risk types were HPV45 in tumor and HPV31 and 56 in urine, which are frequently reported in cervical cancer. High risk HPV types 16, 31, and 45 are more common in colorectal cancer studies than HPV56 (12, 26). Here, the first instance of HPV type 83 being isolated from a colorectal cancer tumor is reported. HPV83 is mainly classified as an intermediate-risk HPV (27), indicating that none of the detected tumor or urine HPVs in this report were low-risk types. Although hr-HPV18 is often found in HPV-related cancers (6), it was not detected in CRC tissue or urine samples in this study. In agreement with present findings, studies performed in Brazil by Damin et al., in Qatar by Fernandes et al., and in the United States by Bernabe-Dones et al. have reported HPV DNA in over 40% of colorectal malignant tissues (10, 12, 28). Conversely, three separate studies conducted in Iran by Nosrati et al., Aghakhani et al., and Taherian et al. did not detect any HPV DNA in their colorectal samples (29–31). In some other studies, the identification rate of HPV DNA was reported to be low (11, 13, 32).

In the current investigation 6 out of the 7 CRC patients who developed metastasis or relapse were confirmed to have HPV16. The detection of HPV16 among the patients with metastastic CRC have been reported before (33). A high rate of HPV16 was found in tumor tissue in this survey, investigating the influence of environmental factors on the transmission and spread of high and low-risk HPV in the human population. Recent research showed the presence of HPV DNA in river in Italy (34) and sewage in Egypt (35), and Uruguay (36). Additionally, oncoviruses HPV DNA was identified in stool samples of patients with diarrhea in Italy (37). So far HPV detection in river, sewage and patients stool were not reported in Iran but it requires further studies.

Alongside cancer diagnosis, metastasis prognosis has been a persistent challenge in cancer management. Circulating or transferring oncoproteins have been implicated in metastasis, as some studies have demonstrated the impact of expression/overexpression of E6 and E7 oncoproteins of HPV type 16 to transform non-invasive cancer cells into invasive or even metastatic ones (38, 39). In addition, few studies have focused on viral oncoproteins in plasma, either free or carried by exosomes (40). Moreover, to the best of our knowledge, the majority of studies on the relationship between exosomes and viruses have been conducted on cell cultures, anti-viral antibodies, or microRNAs (17, 41, 42).

Extracellular vesicles (EVs) including exosomes, have been deemed crucial in the progression of various types of cancers and other pathological conditions over recent years, due to their ability to transfer a variety of oncogenic cargoes (41, 43). HPV16 E7 oncoprotein is one such cargo, as demonstrated by Kannan et al. (41). Considering this, we evaluated the probable circulation of papillomavirus E6 and E7 oncoproteins via exosomes produced by cancer cells. In present study, 15 random serum samples from colorectal cancer patients and 5 serum samples from non-CRC patients were analyzed to detect the presence of the oncoproteins HPV16 E6 and E7 within exosomes. Among these, HPV oncoproteins were detected in two samples from CRC patients. One sample tested positive for both HPV16 E6 and E7 proteins, while the second sample exhibited positivity solely for HPV16 E7 oncoprotein. Interestingly, there were no indications of relapse or metastasis observed in these two CRC patients during the two-year follow-up. According to patients’ claim, one patient had no history of any kind of warts, but the other patient had the history of recovered wart. The current investigation faced some limitations. The small sample size was the first limitation and a large scale study would be a necessity for future investigations. Another pivotal consideration is probing HPV16 E6/E7 mRNAs in tumor tissues or in circulating exosomes to establish a logical relation between the presence of viral DNA and malignancy (44). Moreover, the detection of HPV DNA in bloodstream has been suggested in recent studies as a promising approach for monitoring residual disease or recurrence (45). Analyzing excreted DNA in urine specimens was another way to investigate the various potential routes through which HPV may take advantage to spread itself. The significant proportion (86%) of HPV-positive urine samples tested positive for HPV16, mirroring the findings observed in tumor samples. This parallel positivity in tumor and urine specimens could potentially stem from current or prior infections, supported by existing studies reporting HPV presence in bladder tissue and circulating HPV DNA in the bloodstream (46, 47). Isolating DNA from bladder urine samples may also indicate the potential localization of HPV in upper urinary tract, prostate or probable pre-cancer status (46). This should be considered for further clinical investigations like ultrasound or cystoscopy examinations to ascertain the source of HPV infection.

On the other hand, it is now widely acknowledged that detecting HPV in malignant tissue significantly correlates with a more favorable prognosis and higher survival rate in cervical cancer and HNSCC and should be taken into account in treatment planning for HPV-related cancers (48, 49). To some extent, the current findings align with this concept, given that the majority of HPV positive cases were well to moderately differentiated in histopathological investigation. Future studies are encouraged to explore the concurrent assessment of E6/E7 oncoproteins and corresponding mRNAs alongside cellular oncoproteins/microRNAs, cell-free DNA (cfDNA) and circulating tumor DNA (ctDNA) specific to CRC (50, 51). Employing state-of-the-art kits, equipment or methods tailored for exosome isolation may enhance and fortify these investigations.

The existing data suggests that colorectal cancer screenings should include HPV DNA testing to monitor patient treatment progress. Moreover, given the association of hr-HPVs such as types 16, 18, 31, 33, 45, 58 and other high risk types with various types of cancers such as genital, head and neck and rectal cancers in individuals with different health conditions from healthy to immunocompromised (49, 52), it is imperative to introduce HPV vaccination programs in middle and low-income countries. This is crucial as there is currently no definitive treatment for HPV infections, leading to high hospitalization costs and irreparable burden on infected patients.

## Conclusion

In this study, a noticeable point was an earlier onset of CRC observed, with the youngest patient being 37 years old having HPV16-positive tumor and urine. High HPV DNA detected in CRC tissues and urine CRC patient samples. The parallel analysis of tumor and urine samples for HPV DNA further supports the potential for non-invasive screening tools. The analysis of stool samples of CRC patients to detect HPV DNA could be proposed as additional non-invasive screening method. The detection of HPV16 E6 and E7 oncoproteins in exosomes from routine serum samples underscores the potential of a non-invasive diagnostic method. These outcomes emphesize the potential role of HPV in colorectal cancer development. Therefore, HPV DNA screening is recommended for CRC tissue samples to assess patient survival post-surgery. Given the absence of a definitive treatment for HPV infection, HPV vaccination should be part of preventive measures in low-middle income countries to prevent hr-HPV-related cancers.

## Data availability statement

The original contributions presented in the study are included in the article/Supplementary material, further inquiries can be directed to the corresponding author.

## Ethics statement

The studies involving humans were approved by Approval ethical code IR.AJUMS.REC.1399.817 by the ethics committee of Ahvaz Jundishapur University of Medical Sciences, Ahvaz, Iran. The studies were conducted in accordance with the local legislation and institutional requirements. The participants provided their written informed consent to participate in this study.

## Author contributions

MJ: Conceptualization, Investigation, Methodology, Writing – original draft, Writing – review & editing. SJ: Conceptualization, Methodology, Project administration, Writing – review & editing. MK: Investigation, Writing – review & editing. VK: Investigation, Methodology, Writing – review & editing. AT: Investigation, Writing – review & editing. KA: Data curation, Writing – review & editing. MA: Investigation, Writing – review & editing. MM: Conceptualization, Methodology, Project administration, Supervision, Validation, Writing – original draft, Writing – review & editing.

## Supplementary material

SUPPLEMENTARY FIGURE Graphical abstract for “Time to take HPV infection in colorectal cancer patients more seriously.”
