# Supplementary figures and images for: Time to take HPV infection in colorectal cancer patients more seriously
Source: Front Med (Lausanne). 2024 Jul 10;11:1418359. doi: 10.3389/fmed.2024.1418359 (PMC11266041; doi:10.3389/fmed.2024.1418359)

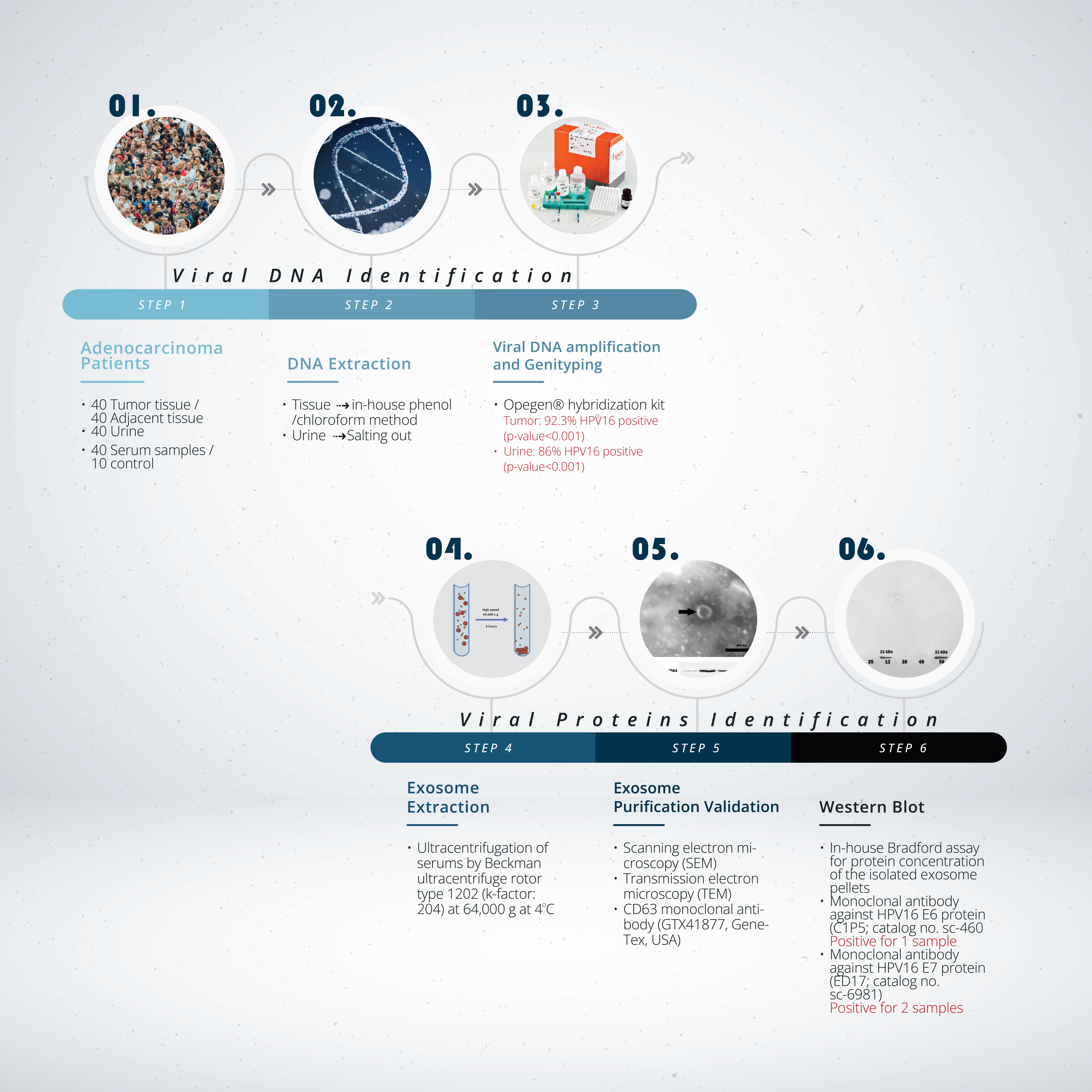

Supplement: SUPPLEMENTARY FIGURE — Graphical abstract for “Time to take HPV infection in colorectal cancer patients more seriously.” [file Image_1.JPEG]
